# Concurrence of leukocyte chemotactic factor 2-associated amyloidosis and autoimmune diseases: A case report

**DOI:** 10.3389/fimmu.2022.966591

**Published:** 2022-08-19

**Authors:** Shuang Wang, Xiao-juan Yu, Dan-yang Li, Jin Xu, Su-xia Wang

**Affiliations:** ^1^ Laboratory of Electron Microscopy, Pathological Center, Peking University First Hospital, Beijing, China; ^2^ Renal Division, Department of Medicine, Peking University First Hospital; Renal Pathological Center, Institute of Nephrology, Peking University; Key Laboratory of Chronic Kidney Disease (CKD) Prevention and Treatment, Ministry of Education of China; Key Laboratory of Renal Diseases, Ministry of Health of China, Beijing, China

**Keywords:** Sjöegren’s syndrome, systemic lupus erythematosus, membranoproliferative glomerulonephritis, leukocyte chemotactic factor 2-associated amyloidosis, kidney biopsy, renal pathology

## Abstract

Leukocyte chemotactic factor 2-associated (ALECT2) amyloidosis is one of the recently reported types of amyloidosis, which is caused by the extracellular deposition of leukocyte chemotactic factor 2 (LECT2). There have not been any reports involving the concurrence of ALECT2 amyloidosis with Sjögren’s syndrome (SS) or systemic lupus erythematosus (SLE)s. Herein, we report a case of a 68-year-old Chinese woman presenting with long duration of sicca symptoms. The clinical evaluation and laboratory findings showed that she had SS overlapped with SLE. Kidney biopsy revealed a membranoproliferative glomerulonephritis (MPGN) with glomerular deposition of dominant IgG3-kappa by immunofluorescene, which was related to SS/SLE. Furthermore, patchy congophilic amyloid deposits in the tubulointerstitium were detected, which were positive for LECT2 protein by immunohistochemical staining and immunoelectron microscopy. This is the first case of ALECT2 amyloidosis that coexisted with SS/SLE, and the causal relationship between ALECT2 amyloidosis and autoimmune diseases remain unclear.

## Introduction

Sjögren’s syndrome (SS) is a chronic autoimmune disorder involving the exocrine glands, predominantly the salivary and lacrimal glands. The main consequence of this inflammation is the development of sicca symptoms, defined as dry eyes and mouth (1). It is noteworthy that SS can be associated with other autoimmune disorders, especially systemic lupus erythematosus (SLE), and they can share many clinical and immunological features.

Leukocyte chemotactic factor 2-associated (ALECT2) amyloidosis, which is characterized by the extracelluar deposition of misfolded proteins derived from leukocyte chemotactic factor 2 (LECT2) and mostly involves the kidney and liver, has recently been reported to be the second or third common type of renal amyloidosis by several studies (2–4). ALECT2 amyloidosis can affect all compartments of the kidney, and the cortical interstitium is the most predominantly involved region (3).

No previous study has reported the association or coexistence between SS/SLE and ALCET2 amyloidosis. Here, we report an SS/SLE patient who had membranoproliferative glomerulonephritis (MPGN) and tubulointerstitial ALECT2 amyloidosis on kidney biopsy. To our knowledge, this is the first case report of MPGN that coexisted with ALECT2 amyloidosis in a patient with SS/SLE.

## Case presentation

A 68-year-old Chinese woman presented with a 20-year history of xerostomia and xerophthalmia. Mild proteinuria was noticed with normal serum creatinine (1.11 mg/dl) 1 month ago when urinary tract infection was suspected; then, she was treated with levofloxacin for 2 weeks. She developed edema, decreased urine output, weakness, and arthralgia 2 weeks before admission, and her serum creatinine was increased to 1.70 mg/dl before admission. She denied malar rash or oral ulcer. Her medical history was significant for 30 years of purpura and 2 months of hypertension. Her mother had end-stage kidney disease of unknown cause.

On physical examination, the patient’s temperature was 37.5°C. Her wrists, metacarpophalangeal joints (MCPs), and shoulders were tender. Some of her MCPs were swollen, and her lower extremities showed severe edema. Muscle power testing score of her upper extremities was normal, but it showed grade 4 for proximal muscles in her legs, which was lower than that of the distal muscles. Other physical examinations were unremarkable.

After admission, urinalysis revealed albumin-dominant proteinuria of 0.97 g/24 h with 30–40 red blood cells per high power field. Urinary N-acetyl-β-(D)-glucosaminidase was 20.4 U/L (0.3–12.0) and α1-microglobulin was 76.40 mg/L (0.00–12.00). Urine myoglobulin was 212.7 ng/ml. Serum albumin was 23 g/L, and serum creatinine was 1.51 mg/dl. The urine output decreased continually, and she developed anuria in 5 days. Complete blood count revealed that white blood cell was 4.7×10^9^/L, hemoglobulin was 102 g/L, platelet count was 181×10^9^/L, mean corpuscular volume was 98 fl (80–100), and reticulocyte count was 3% (1,2). The unconjugated bilirubin was 6.5 μmol/L (1.7–14.0). The hemoglobulin and platelet counts gradually decreased during hospitalization. Direct Coombs test was positive for IgG and negative for C3d. Platelet-associated IgG was normal. Serum lactate dehydrogenase was 469 IU/L (100–240), alanine aminotransferase (ALT) was 135 IU/L (7–40), aspartate aminotransferase was 130 IU/L (13–35), creatine kinase was 1,402 IU/L (2–170), creatine kinase-MB was 13.5 ng/ml (<5), and cardiac troponin I was 0.032 ng/ml (0–0.03). Brain natriuretic peptide was 33 pg/ml (<100). Ultrasound of the kidney showed normal size with increased echogenicity. Echocardiography showed slight thickening of the interventricular septum. CT scan of the lung showed pulmonary edema and mild pleural effusion.

Immunological testing showed that anti-nuclear antibodies were 1:10,000. Anti-dsDNA, anti-SSA, and anti-SSB antibodies were positive. Rheumatoid factor was 1,830 IU/ml (<30). She was positive for type III cryoglobulins in the serum with polyclonal IgG, IgA, IgM, kappa, and lambda. Her serum IgG was 23.70 g/L (7.23–16.85), IgA was 5.44 g/L (0.69–3.82), IgM was 1.40 g/L (0.63–2.77), C3 was 0.30 g/L (0.60–1.50), C4 was 0.04 g/L (0.12–0.36), and C1q was 126.7 mg/L (159.0–233.0). Complement factor H (CFH) was 215.7 μg/ml (247.0–1010.8). Anti-CFH antibodies were negative. Anti-cardiolipin antibodies, lupus anti-coagulants, anti-cyclic citrullinated peptides, anti-neutrophil cytoplasma, anti-Jo-1, and anti-glomerular basement membrane antibodies were all negative. Hepatitis B surface antigen, anti-hepatitis C virus, and anti-human immunodeficiency virus antibodies were all negative. Immunofixation electrophoresis of serum and urine was negative for monoclonal immunoglobulin. PET-CT scan was negative for malignancies. Ultrasound showed no enlarged lymph nodes and normal size spleen.

Ocular dye staining was positive. Salivary gland biopsy showed two dense aggregates of >50 lymphocytes. Her skeletal muscle biopsy revealed that scattered macrophages were infiltrated in the endomysial region, while perifascicular atrophy and rimmed vacuoles were not detected. Based on these findings, a diagnosis of SLE associated with SS was established. Kidney biopsy was performed to evaluate the pathological lesions of the patient.

**Figure 1:**
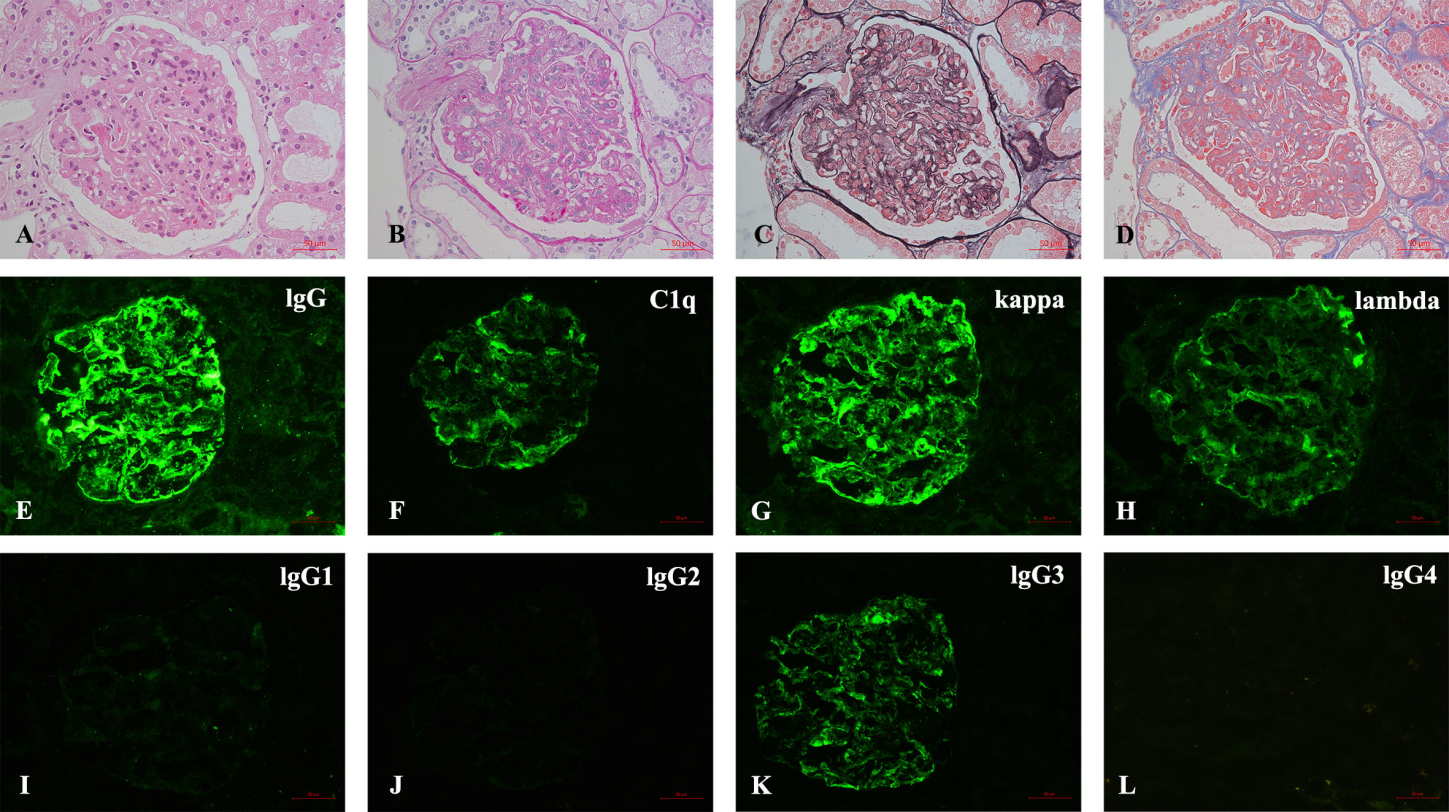
**(A–L)** Pathological findings of renal biopsy. **(A–D)** Light microscopy shows a MPGN pattern of glomerular lesion **(A)**, HE; **(B)**, PAS; **(C)**, PASM; **(D)**, MASSON, 400×). **(E–L)** Immunofluorescence depicts granular mesangial and wall deposits with IgG, C1q, kappa, lambda, IgG3, and IgG2 trace, while IgG1 and IgG4 are negative [**(E–L)**, 400×].

**Figure 2:**
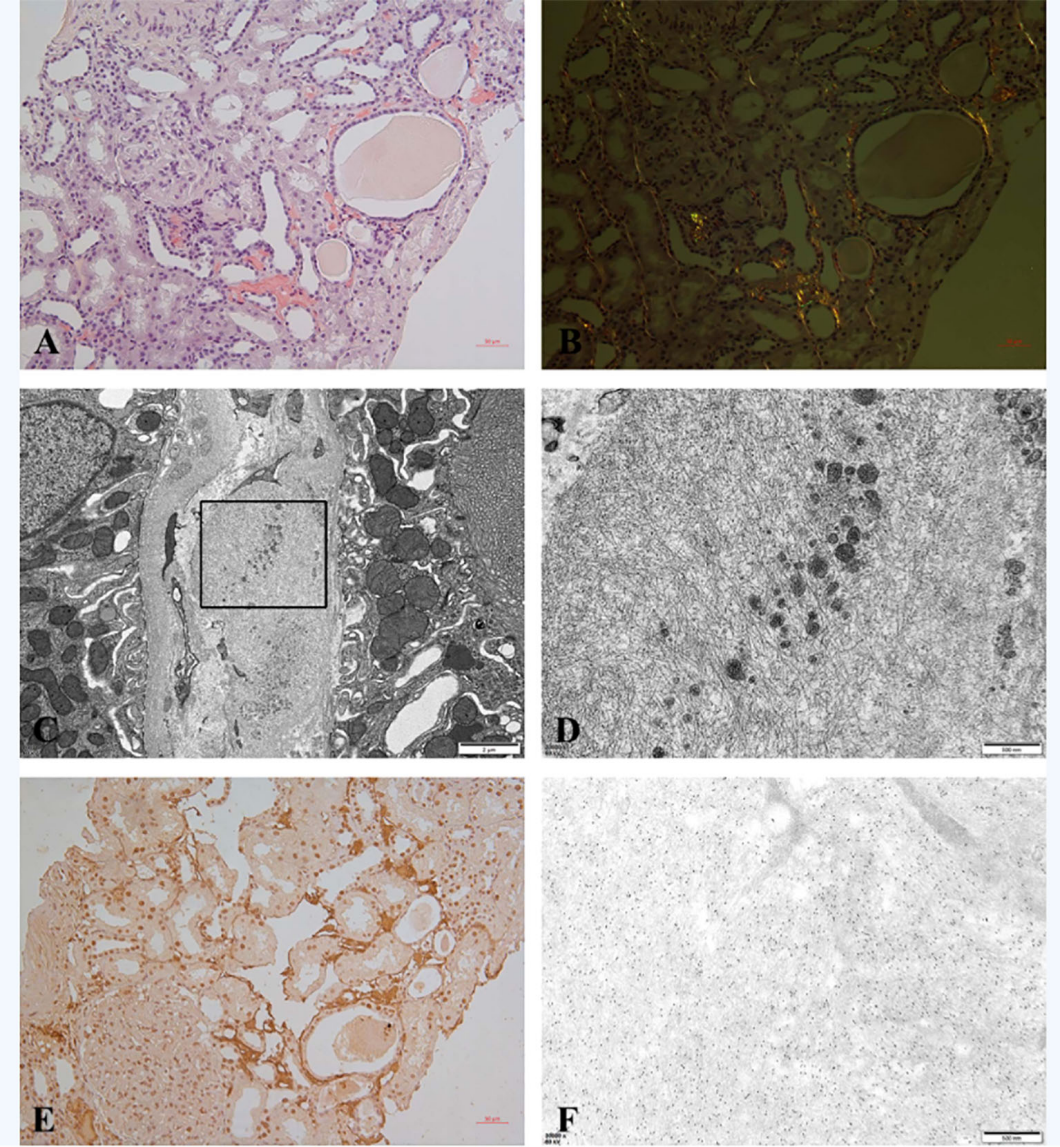
**(A–F)** Amyloid in renal biopsy. **(A)** Congo red staining was positive for amyloid deposits in cortical interstitium (200×). **(B)** Amyloid deposits showed apple-green birefringence under polarized light (200×). **(C)** EM showed randomly arranged fibrils with a diameter of 10 nm in the interstitium (8,000). **(D)** High magnification of selected area in panel **(C)** (35,000×). **(E)** Immunohistochemical staining for LECT2 was positive in amyloid deposits (200×). **(F)** Immune-electron microscopy showed specific labeling of LECT2 in amyloid fibrils (35,000×).

The kidney biopsy findings showed that there were 18 glomeruli including one globally sclerosed in the specimen by light microscopy. Other glomeruli showed a diffuse proliferative glomerulonephritis pattern, which was characterized by severe mesangial cells and matrix proliferation and thickening of glomerular basement membrane with double contours; mesangial and subendothelial eosinophilic deposits were detected. Immunofluorescence on frozen tissue demonstrated granular staining of IgG (3+), IgA (2+), IgM (2+), C3c (2+), C1q (2+), kappa (3+), lambda (+~2+), IgG2 (+), and lgG3 (2+) in the glomerular mesangial area and along the capillary walls. IgG1 and IgG4 were negative (**Figure 1**). Besides, patchy congophilic deposits were found in the cortical interstitium and arteriolar walls, which displayed apple-green birefringence under polarized light. Electron microscopy revealed randomly rigid fibrils with a diameter of 10 nm in the amyloid deposits of cortical interstitium. The amyloid deposits were positive for LECT2 protein by immunohistochemical staining and immune-electron microscopy (**Figure 2**).

She was treated with intravenous methylprednisolone 24 mg daily for 2 weeks, but her condition did not improve; then, methylprednisolone was increased to 40 mg daily. After that, her urine output increased to normal, and she had no fever. Oral prednisolone 50 mg daily was started. Hydroxychloroquine was added at 400 mg daily. However, her serum creatinine and liver and muscular enzymes were still elevated. Monthly intravenous cyclophosphamide 200 mg was added. After 1 month of follow up, she died of multi-organ failure.

## Discussion

SLE is one of the autoimmune disorders mostly associated with SS. In our case, the patient was diagnosed as having SLE associated with SS based on the corresponding classification criteria. She presented with long duration of dry mouth and dry eyes, her ocular dye staining was positive, the biopsy of her salivary gland showed two dense aggregates of >50 lymphocytes, and antibodies to SSA and SSB were both positive. According to the American-European Consensus Group (AECG) Classification Criteria for SS (5), she fulfilled five criteria and was diagnosed as SS. In addition, she had fever, thrombocytopenia, autoimmune hemolysis, musculoskeletal joint involvement, low serum C3 and C4 levels, and positive anti-dsDNA antibodies. Based on the 2019 European League Against Rheumatism/American College of Rheumatology (EULAR/ACR) classification criteria for SLE (6), the diagnosis of SLE was definite. Except for the diagnosis of SS/SLE, the myositis in our case was noted, but she did not fulfill the EULAR/ACR classification criteria for idiopathic inflammatory myopathies (7). We thought that her myositis was related to SS or SLE.

Previous studies showed that SS was frequently associated with SLE, and they had various comparable clinical and immunological features. The prevalence of SS in SLE was 9%–30% (8–10). It has been suggested that patients with SS/SLE were characterized by predominant SS-related features and milder SLE-related features (11), as in our patient whose sicca symptoms preceded the onset of SLE by 20 years.

The occurrence of kidney involvement in SS/SLE varied considerably among the published studies (ranging from 10% to 65%) (8–10, 12), most likely due to the ethnic difference and patient selection criteria. In a large prospective cohort of 259 SS/SLE patients investigated by Baer et al., 29% of these patients were reported to have proteinuria, 23% of them had hematuria, and nephrotic syndrome was identified in 9% of patients (9). In the current case, the patient had mild proteinuria and hematuria, her kidney biopsy further revealed a diffuse membranoproliferative glomerulonephritis (MPGN) pattern, and immunofluorescence showed full-house immunoglobulins and complements deposition. The clinicopathological features of our patient were consistent with SS- and SLE-associated kidney injury.

In addition to SS/SLE-related MPGN, we also noted the dominant deposition of IgG3 along with a stronger kappa light chain in the glomeruli by immunofluorescence. Previous studies found that monoclonal gammopathy could be present in patients with SS and leads to a high risk of lymphoma (13). In the present case, however, there was no evidence of monoclonal gammopathy or lymphoproliferative disorders by clinical manifestations and laboratory examinations. The renal biopsy findings of immunofluorescence revealed a tendency of monoclonal immunoglobulin-related kidney injury, which suggested a latent progression from autoimmune diseases to hematological monoclonal disorders, and an intensive immunosuppressant therapy and cautious follow-up should be guaranteed.

Furthermore, kidney biopsy also revealed the presence of ALECT2 amyloidosis in our patient. The occurrence of renal amyloidosis in patients with SS or SLE remained rare, and they were all amyloid A(AA) amyloidosis in previous reports. There were only two reports about the concomitance of amyloidosis and SS (14, 15). The case number of renal amyloidosis identified in SLE patients was also limited (16–18).

ALECT2 amyloidosis was first reported in 2008 (19) and has become the second or third most common type of renal amyloidosis, following immunoglobulin light chain (AL) amyloidosis and AA amyloidosis (2–4). Most patients with renal ALECT2 amyloidosis presented with mild to moderate proteinuria and varying degrees of renal insufficiency (3, 4, 20). Kidney biopsy showed that all compartments of the kidney can be affected by amyloid deposits, while the cortical interstitium was preferentially involved. In addition, 25%–30% of ALECT2 patients were found to have other concurrent kidney diseases, including diabetic nephropathy, IgA nephropathy, membranous nephropathy, karyomegalic interstitial nephritis, and AA amyloidosis (4, 20–22).

The diagnosis of ALECT2 amyloidosis is based on clinicopathological findings and detection of LECT2 protein in the amyloid deposits. In the current case, no evidence of monoclonal immunoglobulin was indicated, and AL amyloidosis was excluded. By kidney biopsy, amyloid deposits were found in the cortical interstitium and arteriolar walls, while glomerular involvement was more frequent in AL or AA amyloidosis. In addition, the renal amyloid deposits were identified as LECT2 protein by immunohistochemistry and immune-electron microscopy. The ultrastructural immunogold labeling on the amyloid fibrils has been recognized as a highly sensitive and specific method of amyloid typing (23). The common extra-kidney involvement of ALECT2 amyloidosis was the liver, and no biopsy-proven cardiac ALECT2 amyloidosis has been reported. The clinical presentation of ALECT2 patients with liver involvement is the elevation of liver enzymes (24), and our patient had persistent elevation of serum ALT. Therefore, liver involvement due to ALECT2 amyloidosis could not be excluded, but we could not exclude the liver injury secondary to SLE or SS. However, liver biopsy was not done due to low platelet count.

LECT2 is synthesized mainly by hepatocytes; previous studies showed that it was a multifunctional cytokine related to chemotaxis, liver regeneration, immune modulation, bone growth, and glucose metabolism (3). The pathogenesis of ALECT2 amyloidosis is still unknown. The plasma levels of LECT2 tested in patients with ALECT2 amyloidosis were normal (25), and the entire LECT2 protein was found in amyloid fibrils by laser microdissection/mass spectrometry (19). DNA sequencing performed in patients with ALECT2 amyloidosis did not detect any mutation in the *LECT2* gene (4, 19, 25). Because LECT2 has been associated with many immune-mediated process, it is reasonable to hypothesize that the immunopathological responses happening at local tissues can upregulate the expression of LECT2 and eventually lead to ALECT2 amyloidosis. However, the incidental co-occurrence of SS/SLE with ALECT2 amyloidosis could not be excluded yet.

In summary, we report a special case presenting ALECT2 amyloidosis with SS/SLE. To the best of our knowledge, this is the first case report of ALECT2 amyloidosis coexisting with SS/SLE. More investigations are needed to clarify the relationship between the autoimmune diseases and ALECT2 amyloidosis.

## Data availability statement

The original contributions presented in the study are included in the article/supplementary material. Further inquiries can be directed to the corresponding author.

## Ethics statement

The studies involving human participants were reviewed and approved by Peking University First Hospital. The patients/participants provided their written informed consent to participate in this study. Written informed consent was obtained from the individual(s) for the publication of any potentially identifiable images or data included in this article.

## Author contributions

SW, D-yL, and JX performed the experiments of IHC, IEM, and pathology studies. X-jY was in charge of patient care and clinical data collection. SW analyzed data and wrote the original manuscript. S-xW designed this study and revised the manuscript critically for important intellectual content. All authors have read and approved the final manuscript.

## Funding

The work was supported by the National Natural Science Foundation of China (No. 82170724).

## Conflict of interest

The authors declare that the research was conducted in the absence of any commercial or financial relationships that could be construed as a potential conflict of interest.

The reviewer XL declared a shared parent affiliation with the authors to the handling editor at the time of review.

## Publisher’s note

All claims expressed in this article are solely those of the authors and do not necessarily represent those of their affiliated organizations, or those of the publisher, the editors and the reviewers. Any product that may be evaluated in this article, or claim that may be made by its manufacturer, is not guaranteed or endorsed by the publisher.
